# Effect of allogenic demineralized dentin matrix vs. xenograft in maxillary sinus floor augmentation: A randomized split-mouth study

**DOI:** 10.34172/japid.026.3985

**Published:** 2026-05-31

**Authors:** Fatemeh Aghaziarati, Mohammad-Taghi Chitsazi, Leila Roshangar, Amirreza Babaloo, Elnaz Ziaei-Rad

**Affiliations:** ^1^Department of Periodontics, Student Research Committee, Tabriz University of Medical Sciences, Tabriz, Iran; ^2^Dental and Periodontal Research Center, Faculty of Dentistry, Tabriz University of Medical Sciences, Tabriz, Iran; ^3^Department of Anatomical Sciences, Stem Cell Research Center, School of Medicine, Tabriz University of Medical Sciences, Tabriz, Iran; ^4^Department of Periodontics, Faculty of Dentistry, Tabriz University of Medical Sciences, Tabriz, Iran

**Keywords:** Bone regeneration, Demineralized dentin matrix, Dental implants, Sinus floor augmentation

## Abstract

**Introduction:**

Maxillary sinus floor augmentation (MSFA) is commonly required in posterior maxillary atrophy. This study compared allogenic demineralized dentin matrix (ADDM) with a xenograft in MSFA.

**Methods:**

In this randomized split-mouth trial, nine patients underwent bilateral MSFA with ADDM on one side and a xenograft on the other. Cone-beam computed tomography (CBCT) was used to assess bone height and density at baseline and at six months. Core biopsies were obtained for histologic analysis.

**Results:**

Both groups showed significant increases in bone height and density (*P*<0.001). Mean bone density gain was 618.65±232.20 HU (ADDM) vs. 540.87±238.02 HU (xenograft, *P*=0.49). Mean bone height gain was 9.34±1.38 mm (ADDM) vs. 9.12±1.13 mm (xenograft, *P*=0.724). Histology showed viable new bone, with ADDM exhibiting higher collagen fiber density and vascularization.

**Conclusion:**

ADDM showed clinical, radiographic, and histological outcomes comparable to xenograft in MSFA, suggesting its potential as a viable alternative.

## Graphical Abstract

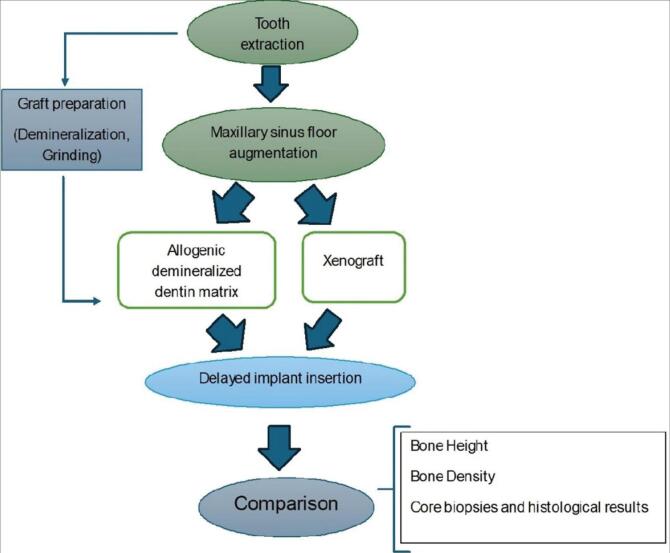

## Introduction

 Dental implant placement in the posterior maxilla is often complicated by sinus pneumatization and alveolar bone resorption after tooth loss, both of which reduce the available bone for implant stability.^1,2^ To overcome these challenges, sinus floor augmentation has become a reliable solution, with high implant survival rates reported in grafted sinuses.^1^ The most established technique is the lateral window approach, first introduced by Tatum and later described by Boyne, in which a lateral bony window is created, the Schneiderian membrane is elevated, and the graft material is placed beneath to preserve space for bone regeneration.^1,3-5^

 Demineralized dentin matrix (DDM) contains bioactive factors, including TGF-β, IGFs, BMPs, bFGF, and non-collagenous proteins like phosphoproteins.^6^ The demineralization method, chemical agent, and exposure duration affect the organic matrix structure and regenerative potential, with agents such as EDTA, HCl, and nitric acid commonly used.^7^ Partially demineralized dentin preserves more growth factors and shows greater regenerative potential than fully demineralized dentin,^8^ as it decreases calcium and phosphorus content while increasing BMP-2 bioavailability.^9,10^ Autogenous dentin grafts prepared with the Tooth Transformer device have been successfully applied in socket preservation and maxillary sinus augmentation, integrating completely with host tissue without inflammatory response.^11,12^

 DDM has osteoinductive properties, promoting mesenchymal cell differentiation into chondroblasts and osteoblasts, contributing to new bone formation.^13^ Early studies by Urist and Yeomans (1967) showed that DDM releases BMPs, inducing bone formation.^14-16^ Um^17^ confirmed that dentin can carry BMP-2 while retaining osteoinductive potential after acid treatment.^17^ Acid-treated dentin also releases calcium and develops a porous structure that enhances fibrovascular organization and osteogenesis. Rijal and Shin^18^ reported that demineralized dentin has superior bone regeneration potential compared to mineralized dentin.^18^

 DDM has been investigated as a graft material for socket preservation, guided bone regeneration, and the treatment of periodontal defects.^19,20^ While autogenous bone remains the “gold standard” due to its osteogenic and osteoinductive properties,^21,22^ dentin offers a practical alternative, being compositionally similar to alveolar bone and containing hydroxyapatite, collagen types I, III, and V, and non-collagenous proteins.^23-25^

 Autogenous DDM (Auto-DDM) has demonstrated efficacy in bone regeneration but remains limited by unpredictable availability and case-dependent quality. To overcome these drawbacks, allogenic DDM (ADDM) has been proposed. Early in vivo studies by Urist and Bang (1967) demonstrated that DDM induces bone formation.^26^ Subsequent experimental work in the 1970s and 1980s confirmed that ADDM possesses excellent bone-forming potential without eliciting immune or antigenic reactions.^27,28^ Clinical trials with frozen allogenic dentin have shown significant regenerative outcomes in periodontal and maxillofacial defects.^29^ Kim et al.^30^ also reported successful alveolar bone augmentation in 18 patients using ADDM, without immunologic complications and with results comparable to Auto-DDM.^30,31^

 In 2017, the first randomized controlled clinical trial compared dentin allograft (DA) and whole-tooth allograft (WTA) with freeze-dried bone allograft (FDBA) for alveolar ridge preservation in 15 patients. The DA and WTA groups demonstrated the least reduction in ridge height and width, as well as greater new bone formation.^32^

 Although Auto-DDM has demonstrated efficacy in bone regeneration, its availability is limited and case-dependent. ADDM has been proposed to overcome these limitations, yet clinical evidence evaluating its effectiveness in MSFA remains scarce. Moreover, no randomized split-mouth clinical trials have directly compared ADDM with xenograft. Therefore, the primary objective of this study was to evaluate the clinical, radiographic, and histologic healing outcomes of ADDM compared with xenograft in MSFA.

## Methods

###  Study Design and Population

 This double-masked, randomized, split-mouth clinical trial was conducted in 2022‒2023 at the Faculty of Dentistry, Tabriz University of Medical Sciences. Nine patients aged 31–65 years (mean ± SD: 48.2 ± 10.7 years), partially or completely edentulous in the posterior maxilla with residual bone height ≤ 5 mm, were enrolled. Patients with sinus pathology, systemic conditions affecting bone healing, smoking, pregnancy, or uncontrolled systemic diseases were excluded. Written informed consent was obtained from all the participants. The study protocol was approved by the Institutional Ethics Committee (Approval No.: IR.TBZMED.DENTISTRY.REC.1403.046).

###  Preparation of ADDM

**Figure 1:**
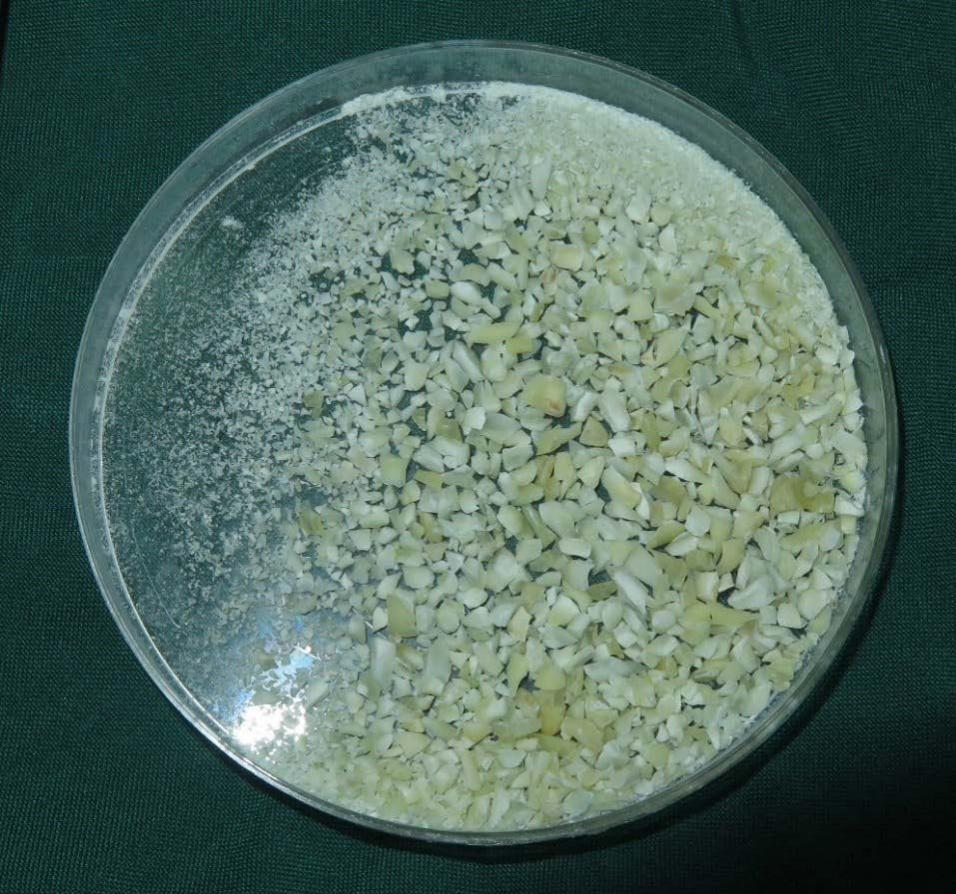
ADDM: allogenic demineralized dentin matrix

 Extracted hopeless teeth (due to advanced periodontal disease or non-functional third molars) were cleaned of soft tissue remnants, cementum, and pulpal tissue. Enamel was removed with a high-speed bur. Dentin was crushed into 1–1.5-mm particles and washed with sterile saline. Particles were demineralized using 2% nitric acid for 10 minutes, neutralized with 0.1-M Tris-HCl for 10 minutes, and acellularized using trypsin, raffinose, and sucrose solutions. Final sterilization was achieved with UV irradiation after triple washing in PBS containing penicillin/streptomycin (Figure 1).

###  Surgical Procedure

**Figure 2:**
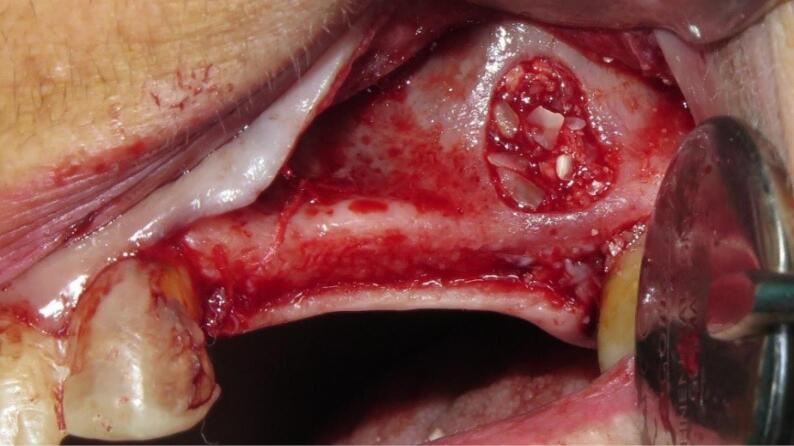
Application of allogenic demineralized dentin matrix (ADDM) for sinus cavity filling

**Figure 3:**
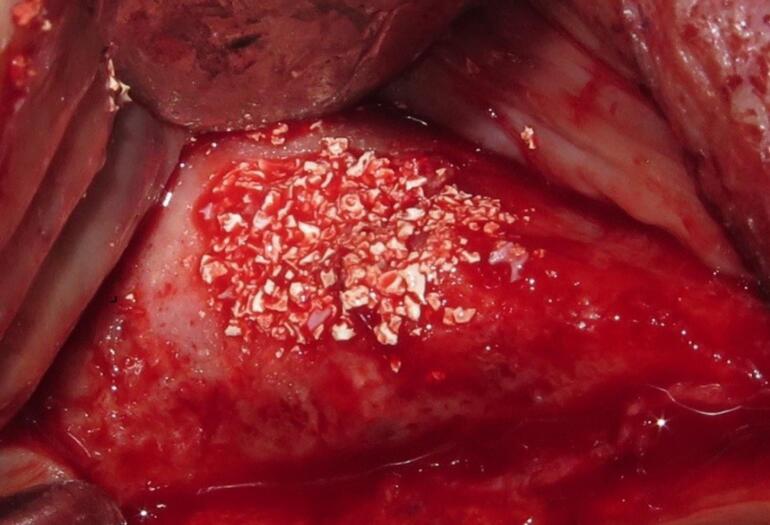
Application of xenograft for sinus cavity filling

 MSFA was performed using the lateral window approach. A mid-crestal incision with or without a buccal releasing incision was made, and a bony window was prepared on the lateral sinus wall with a round bur. After carefully elevating the Schneiderian membrane, 2 mL of ADDM was placed in the test sinus (Figure 2), while 2 mL of xenograft (Bone + B, Nova Teb Pars, Iran), with particle sizes of 0.25‒1 mm, was placed in the contralateral sinus as a control (Figure 3). Postoperative medications included amoxicillin-clavulanic acid (625 mg tid for 7 days) and ibuprofen (400 mg tid for pain control). 0.2% chlorhexidine mouthrinse was prescribed twice daily for 7 days.

###  Radiographic Analysis

**Figure 4:**
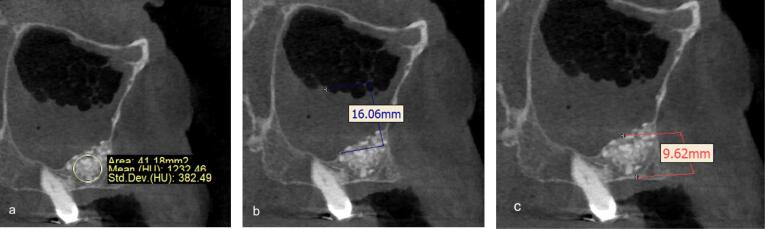
Measurement using Mimics software: a) Bone density. b) Sinus membrane thickness. c) Bone height

 Preoperative and 6-month postoperative CBCT scans were obtained at the same radiology center using identical imaging parameters to ensure standardization. Bone height (mm) and density (Hounsfield Units, HU) measurements were performed using Mimics 21.01 software. All measurements were performed by a calibrated examiner who was blinded to the group allocation to minimize assessment bias. Calibration was conducted before the study by having the examiner measure a subset of scans twice, one week apart, to ensure consistent measurements. The results obtained from both groups were then compared statistically (Figure 4).

###  Histologic Evaluation 

 At six months, trephine core biopsies (4 mm in diameter) were obtained during implant placement. The samples were fixed in 10% formalin, decalcified, embedded in paraffin, sectioned, and stained with Hematoxylin and Eosin and Masson’s Trichrome. Histomorphometric analysis was performed using Motic Image 2 software. The area of newly formed viable bone and the residual graft material was measured in mm^2^. Collagen fiber organization and vascularization were also assessed qualitatively.

###  Statistical Analysis 

 Data normality was assessed using the Kolmogorov-Smirnov test. Paired t-tests were used for inter-group comparisons of bone height and density, while independent t-tests compared differences between groups. Exact *P*-values were reported, with significance set at *P* < 0.05. All the analyses were performed using SPSS 26 (IBM Corp., Armonk, NY, USA).

## Results

###  Radiographic Results

 Both groups demonstrated significant postoperative increases in bone density and height compared with baseline (*P* < 0.001).

###  Bone Density

 The mean bone density at six months was 816.35 ± 282.36 HU in the control group and 949.92 ± 271.83 HU in the test group. In both groups, a significant difference was observed between the bone density values before surgery and those measured six months postoperatively (Table 1).

**Table 1:** Comparison of bone density values before and 6 months after surgery (paired-samples t-test)

**Variable**	**N**	**Mean (HU)**	**SD**	**T**	***P*** **-value**
ADDM (Pre)	9	331.27	97.42		
ADDM (Post)	9	949.93	271.83	-7.99	0.00
Xenograft (Pre)	9	275.48	110.98		
Xenograft (Post)	9	816.36	282.36	-6.82	0.00

Paired t-test was used for within-group comparisons.

 To compare the bone density formed between the test and control groups at six months, the difference between preoperative and six-month postoperative density values was used. The mean density difference was 540.87 ± 238.02 HU in the control group and 232.20 ± 618.65 HU in the test group. Although the mean density difference was higher in the test group, the paired t-test showed no statistically significant difference between the test and control groups (*P* = 0.49) (Table 2).

**Table 2:** Comparison of the difference in bone density between the ADDM and xenograft group (Independent t-test)

**Group**	**N**	**Mean difference (HU)**	**SD**	**T**	***P*** **-value**
ADDM	9	618.65	232.20	-0.70	0.49
Xenograft	9	540.88	238.02		

Independent t-test was used for between-group comparisons.

###  Bone Height

 The mean bone height at six months was 12.08 ± 1.16 mm in the control group and 11.89 ± 2.24 mm in the test group. In both the test and control groups, a significant difference was observed between bone height before surgery and that measured six months postoperatively (Table 3).

**Table 3:** Comparison of bone height before and 6 months after surgery (paired-samples t-test)

**Variable**	**N**	**Mean (mm)**	**SD**	**T**	***P*** **-value**
ADDM (Pre)	9	2.55	1.11		
ADDM (Post)	9	11.89	2.24	-20.26	0.00
Xenograft (Pre)	9	2.96	0.74		
Xenograft (Post)	9	12.09	1.17	-24.03	0.00

Paired t-test was used for within-group comparisons.

 To compare the bone height formed between the test and control groups at six months, the difference between preoperative and six-month postoperative height values was used. The mean height difference was 9.12 ± 1.13 mm in the control group and 9.34 ± 1.38 mm in the test group. Although the mean height difference was slightly higher in the test group, the paired t-test showed no statistically significant difference between the test and control groups (*P* = 0.72) (Table 4). In other words, the increase in bone height did not differ significantly between the two groups.

**Table 4:** Comparison of the difference in bone height between the ADDM and Xenograft group (Independent t-test)

**Group**	**N**	**Mean difference (mm)**	**SD**	**T**	***P*** **-value**
ADDM	9	9.34	1.38	-0.36	0.72
Xenograft	9	9.12	1.13		

Independent t-test was used for between-group comparisons.

###  Histological Results

**Figure 5:**
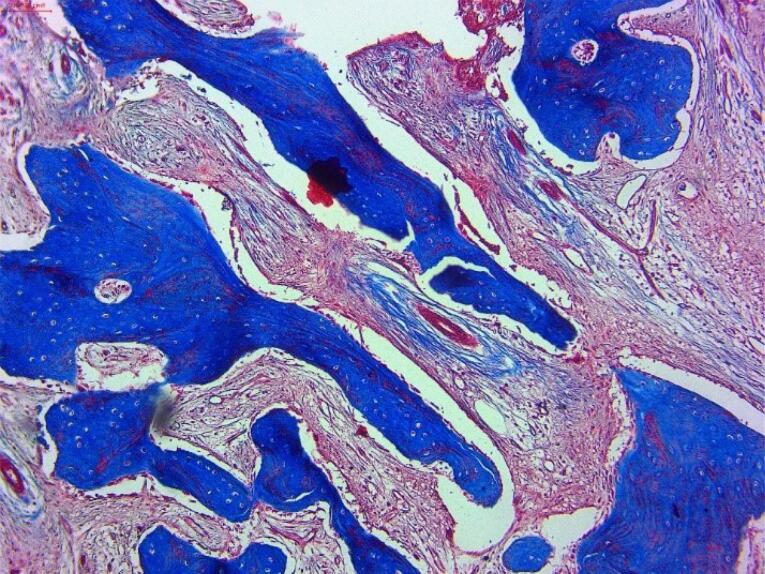
Viable bone formation in the allogenic demineralized dentin matrix (ADDM) group

**Figure 6:**
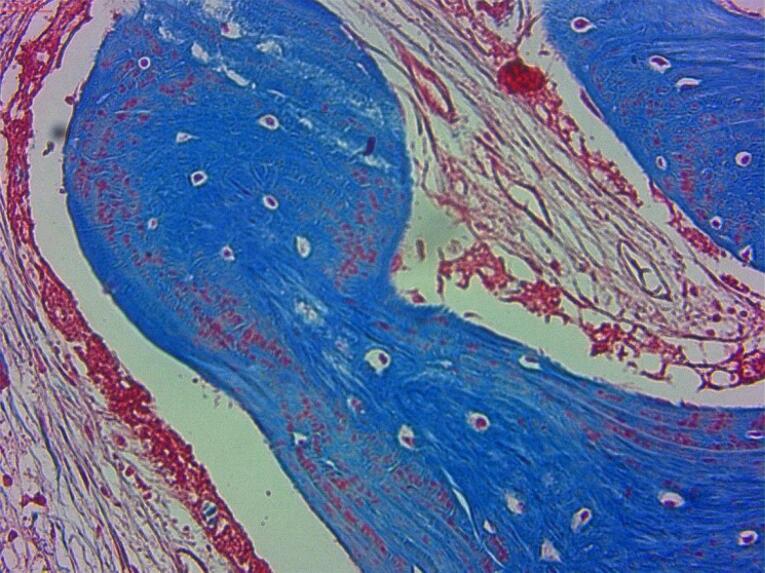
Residual allogenic demineralized dentin matrix (ADDM) integrated with the new bone

 Both groups exhibited viable bone formation characterized by osteocytes within lacunae and trabecular organization. In the ADDM group, more organized collagen fibers and greater vascularization were observed compared with the xenograft group (Figure 5); however, these did not translate into a clinically significant difference in the primary clinical outcomes of bone height and bone density gain between the groups. Residual dentin particles were occasionally detected in the ADDM group and appeared to be fully integrated with newly formed bone without evidence of inflammatory response (Figure 6). In contrast, xenograft samples also demonstrated new bone formation, although with relatively fewer collagen bundles and less vascularization.

## Discussion

 The maxillary sinus cavity represents a confined defect that is not a natural site for spontaneous bone formation; therefore, it has been widely used as a reliable model for evaluating bone grafting materials. Therefore, in the present study, the maxillary sinus cavity was selected as the model to assess the regenerative potential of two different bone graft materials.^33^ This split-mouth randomized clinical trial compared ADDM with xenograft in MSFA. Both grafting materials resulted in significant increases in bone density and bone height six months postoperatively, confirming their effectiveness for sinus augmentation procedures. Although ADDM showed higher mean values in both parameters, statistical analysis revealed no significant differences compared to the xenograft.

###  Bone Density

 In this study, both the xenograft and ADDM groups exhibited a significant increase in bone density from baseline to six months postoperatively (*P* = 0.0). The mean increase in bone density in the ADDM group was 618.65 ± 232.20 HU, while in the xenograft group, it was 540.87 ± 238.02 HU. Although the mean bone density increase in the ADDM group was slightly higher, this difference was not statistically significant (*P* = 0.49), indicating a comparable performance of both materials in improving the quality of regenerated bone. These findings are consistent with previous clinical studies evaluating tooth-derived graft materials. Kim et al.^34^ (2013) evaluated bone regeneration nine months after MSFA using autogenous tooth-derived graft material (AutoBT) through micromorphometric and histological analyses. Micro-CT analysis revealed a total bone volume (including graft and newly formed bone) of 76.45%, with newly formed bone accounting for 45.4% of the total volume. The mean Hounsfield Unit (HU) of new bone was 1164.69, indicating adequate bone density.^34^

 Jun et al.^23^ (2014) reported similar results. Bone density was assessed using HU in the Bio-Oss and AutoBT groups. Preoperatively, the alveolar bone density was 421.73 HU in the Bio-Oss group and 380.28 HU in the AutoBT group, both classified as D3. Nine months postoperatively, mean bone density increased to 968.15 HU and 981.80 HU, respectively, corresponding to D2 classification, with no statistically significant difference between the groups (*P* = 0.36).^23^

 Overall, the significant increase in bone density reflects successful bone regeneration with tooth-derived grafts, providing performance comparable to that of Bio-Oss. Enhanced bone quality and density are crucial for improving dental implant stability and ensuring long-term surgical success.

 Collectively, these results suggest that tooth-derived graft materials provide bone quality comparable to xenografts, supporting their clinical reliability for implant site development.

###  Bone Height

 Bone height was evaluated by comparing the preoperative and six-month postoperative measurements. The mean height increase was 9.12 ± 1.13 mm in the xenograft group and 9.34 ± 1.38 mm in the ADDM group. Although the ADDM group showed a slightly higher mean increase, the difference between groups was not statistically significant (*P* = 0.72).

 Minetti et al.^35^ (2019) evaluated autogenous tooth matrix grafts in patients with posterior maxillary atrophy. The residual ridge height increased significantly from 2.04 ± 5.22 mm to 2.83 ± 14.72 mm after six months (*P* < 0.05).^35^

 Jun et al.^23^ reported similar results, with comparable increases in bone height in both the Bio-Oss and AutoBT groups after MSFA.^23^ Kim et al.^36^ (2016) demonstrated a significant increase in bone height using demineralized tooth blocks combined with platelet-rich plasma (PRP), with a mean increase from 0.8 ± 3.1 mm to 13.8 ± 2.5 mm after six months (*P* < 0.001).^36^

 Paetnukroh et al.^37^ reported average residual bone heights of 1.2 ± 3.5 mm (tooth matrix) and 1.1 ± 3.4 mm (deproteinized bovine bone mineral, DBBM) with no significant differences. After MSFA, the bone height increased significantly in both groups (2.3 ± 12.8 mm and 2.5 ± 13.1 mm, respectively; *P* < 0.001).^37^

 Jeong and Lee^38^ evaluated three graft materials (AutoBT, DFDBA, and DBBM) and observed no statistically significant differences in bone height at six months postoperatively (2.22 ± 13.56 mm, 2.18 ± 13.90 mm, and 2.15 ± 13.67 mm, respectively; *P* < 0.05),^38^ confirming similar efficacy across the materials.

 Fattouh and Ali^39^ (2018) reported a mean residual bone height of 2.04 ± 5.22 mm preoperatively, which increased to 2.83 ± 14.72 mm at six months postoperatively using freshly extracted autogenous tooth grafts (*P* < 0.001).^39^ These findings demonstrate the successful regeneration and volume preservation of sinus bone, providing optimal conditions for implant placement.

###  Maxillary Sinus Membrane Thickness and Biocompatibility

 In the present study, despite the absence of clinical symptoms, membrane thickness on radiographs was slightly greater in the ADDM group than in the xenograft group. This finding warrants cautious interpretation and highlights the need for longer follow-up periods to monitor potential inflammatory responses. Similar transient membrane thickening has been reported by Jun et al.,^23^ with resolution observed over time, suggesting that such changes may represent a temporary postoperative response rather than true pathological inflammation.^23^

###  Histological Evaluation

 In an animal study by Lee et al.^40^ (2013), autogenous tooth grafts placed in the maxillary sinus led to substantial, cohesive new bone formation, comprising cortical and trabecular bone with well-defined vascular networks. Image analyses indicated a significantly higher percentage of new bone in the treatment group (*P* < 0.05), with reduced inflammatory cell infiltration, suggesting good tissue tolerance and an absence of a severe immune response. Tooth graft particles integrated well with new bone, supporting complete endosteal osteogenesis.^40^

 Fattouh and Ali^39^ (2018) reported 42–48% new bone formation with minimal residual graft material ( < 25%), demonstrating favorable resorption and replacement by host bone.^39^ Similarly, Xu et al.^41^ (2018) observed higher percentages of new bone formation in human tooth grafts compared to Bio-Oss, with faster resorption and minimal inflammation.^41^

 Limitations of the present study include a small sample size and a short follow-up period. Future studies with larger cohorts and longer follow-up are recommended to evaluate implant survival and long-term outcomes. Additionally, further clinical studies are suggested to compare the biocompatibility of allogenic tooth grafts processed through different preparation methods in MSFA procedures.

## Conclusion

 ADDM demonstrated outcomes similar to those of the xenograft in MSFA, including clinical, radiographic, and histological outcomes. Both grafting materials significantly increased bone density and height after six months. Histological evaluation showed enhanced collagen organization and angiogenesis in ADDM compared with xenograft; however, no statistically significant differences were observed in the primary clinical outcomes. These findings suggest that ADDM is a viable alternative to xenograft for maxillary sinus augmentation procedures.

## Competing Interests

 The authors declare no conflicts of interest related to this study.

## Data Availability

 The datasets used and/or analyzed during the current study are available from the corresponding authors upon reasonable request.

## Ethical Approval

 This study was approved by the Institutional Ethics Committee of Tabriz University of Medical Sciences (Approval No. IR.TBZMED.DENTISTRY.REC.1403.046).
